# An *Infectious Bursal Disease Virus* (IBDV) Reverse Genetics Rescue System and Neutralization Assay in Chicken B Cells

**DOI:** 10.1002/cpz1.639

**Published:** 2023-01-09

**Authors:** Vishwanatha R. A. P. Reddy, Salik Nazki, Amin Asfor, Andrew J. Broadbent

**Affiliations:** ^1^ The Pirbright Institute Woking UK; ^2^ Department of Animal and Avian Sciences University of Maryland College Park Maryland; ^3^ Department of Comparative Biomedical Sciences, Section Infection and Immunity, School of Veterinary Medicine, Faculty of Health and Medical Sciences University of Surrey Guilford UK

**Keywords:** chicken B‐cells, infectious bursal disease virus, neutralization assay, reverse genetics

## Abstract

Infectious bursal disease virus (IBDV) is a major threat to the productivity of the poultry industry due to morbidity, mortality, and immunosuppression that exacerbates secondary infections and reduces the efficacy of vaccination programs. Field strains of IBDV have a preferred tropism for chicken B cells, the majority of which reside in the bursa of Fabricius (BF). IBDV adaptation to adherent cell culture is associated with mutations altering amino acids in the hypervariable region (HVR) of the capsid protein, which affects immunogenicity and virulence. Until recently, this has limited both the application of reverse genetics systems for engineering molecular clones, and the use of in vitro neutralization assays, to cell‐culture‐adapted strains of IBDV. Here, we describe the rescue of molecular clones of IBDV containing the HVR from diverse field strains, along with a neutralization assay to quantify antibody responses against the rescued viruses, both using chicken B cells. These methods are readily adaptable to any laboratory with molecular biology expertise and negate the need to obtain wild‐type strains. © 2023 The Authors. Current Protocols published by Wiley Periodicals LLC.

**Basic Protocol 1**: A chicken B‐cell rescue system for IBDV

**Basic Protocol 2**: A chicken B‐cell neutralization assay for IBDV

## INTRODUCTION

*Infectious bursal disease virus* (IBDV) belongs to the family Birnaviridae and the genus *Avibirnavirus*. IBDV is endemic worldwide, threatening the poultry industry in almost every country surveyed. In addition to being responsible for disease in birds that can be severe, leading to significant economic losses globally, IBDV‐mediated immunosuppression hinders the efficacy of other vaccination programs and exacerbates secondary infections, some of which are of zoonotic importance. The virus has a double‐stranded RNA genome encoding two segments, A and B. Segment A has two partially overlapping open reading frames (ORF), in which ORF A1 encodes the non‐structural viral protein VP5 (Mendez et al., 2017) and ORF A2 encodes a large polyprotein that undergoes cleavage by the protease VP4 to yield the proteins VP2, VP4, and VP3 (Lejal, Da Costa, Huet, & Delmas, 2000). VP2 is the capsid protein, and VP3 is a scaffold protein between the genome and the capsid (Delgui, Ona, et al., 2009; Luque et al., 2009). Segment B has one ORF that encodes the RNA‐dependent RNA polymerase (VP1) enzyme (Garriga et al., 2007).

IBDV is a non‐enveloped virus with an icosahedral capsid formed from the VP2 protein. The VP2 capsid is known to be an important immunodominant protein of IBDV and is the major target of neutralizing antibodies, which are thought to be the main correlates of protection. Within the VP2 capsid, there is a “hypervariable region” (HVR), which is subject to the most intense immune selection pressure and antigenic drift. IBDV strains are classified into eight genogroups based on the sequence diversity of the HVR, named as genogroups A1‐A8 (Islam et al., 2021; Michel & Jackwood, 2017).

IBDV vaccine failures worldwide have been attributed to the emergence of strains with mutations affecting the HVR (Aliyu, Hair‐Bejo, Omar, & Ideris, 2021; Fan et al., 2019; Islam et al., 2021; Morla, Deka, & Kumar, 2016). Therefore, it is important to understand how the IBDV HVR sequence relates to antigenicity. Reverse genetics provides an opportunity to study this in more detail, as individual point mutations can be introduced into the HVR and their effects on antigenicity determined. However, field strains of IBDV have a preferred tropism for chicken B cells, and these field IBDVs do not replicate well in immortalized adherent cell lines without prior adaptation. Unfortunately, adapting IBDV to replicate in immortalized cell culture in the laboratory is associated with mutations in the HVR that can change antigenicity (Lim, Cao, Yu, & Mo, 1999; Mundt, 1999; van Loon, de Haas, Zeyda, & Mundt, 2002). Therefore, it is necessary to use chicken B cells to rescue molecular clones of field strains and to quantify antibody responses against IBDV field strains.

## STRATEGIC PLANNING

### Planning for Basic Protocol 1

First, reverse genetics plasmids must be designed and synthesized. Typical reverse genetics plasmids contain an insert consisting of the self‐cleaving hammerhead ribozyme followed by the 5′ noncoding region, the coding region of either segment A or B, the 3′ noncoding region, and a self‐cleaving hepatitis delta ribozyme, in an expression vector under the control of the cytomegalovirus (CMV) promoter. Typically, we use pSF‐CAG‐KAN as the vector. Inserts may be commercially synthesized and then cloned out of the commercial vector into the multiple cloning site of pSF‐CAG‐KAN using suitable restriction enzymes. Second, although we have used the chicken B‐cell line DT40 in our studies, it should be possible to electroporate primary chicken bursal cells with reverse genetics plasmids to rescue a molecular clone of IBDV; however, the electroporation settings will need to be optimized to maximize the uptake of the plasmid while minimizing cell death. Primary bursal cells can also be used to titrate IBDV (Dulwich, Asfor, Gray, Nair, & Broadbent, 2018; Soubies et al., 2018) and should be appropriate for use in neutralization assays. Moreover, this protocol is applicable to different IBDV strains and can be used to rescue lab‐adapted IBDV strains or field strains.

### Biosafety Considerations

*CAUTION*: Approval should be obtained from the institutional biosafety committee (IBC) before commencing the work. All work should be conducted in a microbiological safety cabinet approved to BSL2 standards. Personnel should wear appropriate personal protective equipment (PPE), such as a laboratory coat and nitrile gloves. IBDV is inactivated by 1% Virkon ® S (Antec DuPont, cat. no. 130000014173) applied for 10 min, so liquid waste should be treated with Virkon, prepared at a final concentration of at least 1%. Solid waste and surfaces should be decontaminated with 1% Virkon for at least 10 min, and solid waste should be autoclaved before disposal. Personnel should be adequately trained in virus handling and waste disposal.

## Basic Protocol 1 A CHICKEN B‐CELL RESCUE SYSTEM FOR IBDV

**Figure 1:**
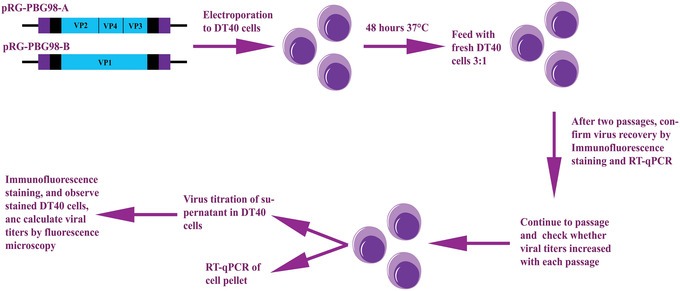
Overview of the chicken B‐cell rescue system for IBDV. To rescue IBDV strains, electroporate reverse genetics plasmids for segments A and B into DT40 cells. Reverse genetics plasmids, in this example for the IBDV strain PBG98 (pRG‐PBG98‐A and pRG‐PBG98‐B), are constructed to contain an insert consisting of the self‐cleaving hammerhead ribozyme (purple box) followed by the 5′ noncoding region (black box), the coding region of either segment A or B, the 3′ noncoding region (black box), and a self‐cleaving hepatitis delta ribozyme (purple box), in an expression vector under the control of the CMV promoter. Then, plasmids pRG‐PBG98‐A and pRG‐PBG98‐B electroporated into DT40 cells and incubated for 48 hr at 37°C. Next, cells are passaged by the addition of fresh DT40 cells to the cultures in a 3:1 ratio and the cells are incubated for 72 hr at 37°C. After two such passages, an aliquot of the cell suspension is harvested, and virus rescue is confirmed through immunofluorescence staining of DT40 cells and RT‐qPCR quantification of the cell pellet. Finally, the cells are passaged further and assessed to determine whether viral titers have increased with each passage by titrating the supernatant for viral titers and quantifying the cell pellet by RT‐qPCR. The figure was created using templates from the Motifolio toolkit (Motifolio Inc., Ellicott City, MD, USA).

The rescue of a molecular clone of IBDV in the chicken B‐cell line DT40 involves the electroporation of the DT40 cells with two reverse genetics plasmids, one encoding segment A and one encoding segment B. To evaluate the success of the rescue, cells should be fixed and stained with a primary antibody against IBDV and then a secondary antibody conjugated to a fluorophore to identify cells that are positive for virus antigen. Viral replication should be quantified by reverse transcription quantitative polymerase chain reaction (RT‐qPCR). Quantification of virus should be performed over multiple passages to confirm a rising viral titer. An outline of the protocol is shown in Figure 1.

### Materials

Synthesized segment A and B from IBDV named as pIBDV‐A and pIBDV‐B (Gene Art; Thermo Fisher Scientific)
Nuclease‐free water (Ambion, cat. no. AM9937)
*Escherichia coli* DH5α (New England Biolabs: NEB^®^ 5‐alpha competent *E. coli*; C2984H, high efficiency)
SOC medium (New England Biolabs: B9020S)
LB agar plate supplemented with 5 μg/ml ampicillin
LB broth supplemented with 5 μg/ml ampicillin (Gibco, cat. no. 10855‐021)
Maxi plasmid purification kit (Qiagen, cat. no. 12162)
KpnI‐HF^®^ restriction enzyme (New England Biolabs, cat. no. R3142S)
NheI‐HF^®^ restriction enzyme (New England Biolabs, cat. no. R3131S)
1% (w/v) agarose/TBE gel
6× DNA loading dye (New England Biolabs, B7024A, 10089404)
10× TBE electrophoresis buffer (Thermo Scientific, 00786866)
1 kb plus DNA ladder (New England Biolabs, N0550S, 10092850)
GFX PCR DNA and Gel Band Purification Kit (Illustra, cat. no. 28903470)
pSF‐CAG‐KAN vector (Sigma‐Aldrich, cat. no. OGS505)
T4 DNA ligase (New England Biolabs, cat. no. M0202S)
Ice (Scotsman)
Mini plasmid preparation kit (Qiagen, cat. no. 27106)
pSF‐CAG‐KAN primers:
  pSF‐CAG‐KAN vector forward primer: 5′‐CTACCATCCACTCGACACACC‐3′ (10 μM stock; Integrated DNA Technologies)
  pSF‐CAG‐KAN vector reverse primer: 5′‐GTTGTGGTTTGTCCAAACTCATCA‐3′(10 μM stock; Integrated DNA Technologies)
Opti‐MEM medium (Gibco, cat. no. 31985070)
Complete DT40 medium (see recipe)
DT40 cells (chicken bursal lymphoma transformed B‐cell line; ATCC, cat. no. CRL‐2111) grown in 75‐cm^2^ (T75) cell culture flasks (TPP, 90076, cat. no. 20210105)
10 mM 4dNTP mix (dATP, dCTP, dGTP, dTTP)
GoTaq G2^®^ Hot Start Master Mix (Promega, cat. no. M7422)
4% (v/v) paraformaldehyde (Santa Cruz Biotechnology, cat. no. sc‐281692)
Triton™ X‐100 (Scientific Laboratory Supplies, cat. no. T8787)
Phosphate‐buffered saline (PBS), pH 7.4
4% BSA in PBS
Primary antibody: Mouse monoclonal raised against IBDV VP3 protein (Pirbright Institute; available on request)
Secondary antibody: Goat anti‐mouse antibody conjugated to Alexa Fluor 488 or 568 (Thermo Fisher Scientific, cat. no. A11029/A11004)
4′,6′‐Diamidino‐2‐phenylindole (DAPI; Thermo Fisher Scientific, cat. no. D1306)
RNeasy mini kit (Qiagen, cat. no. 74104)
SuperScript III Reverse Transcriptase (Thermo Fisher Scientific, cat. no. 18080044)
Taqman Universal PCR Master Mix (Applied Biosystems, cat. no. 4352042)
IBDV primers:
  IBDV RT‐qPCR forward primer 5′‐GAGGTGGCCGACCTCAACT‐3′ (10 μM stock; Integrated DNA Technologies)
  IBDV RT‐qPCR reverse primer 5′‐GCCCGGATTATGTCTTTGAAG‐3′ (10 μM stock; Integrated DNA Technologies)
  IBDV Taqman Probe 5′‐ FAM‐CCCCTGAAGATTGCAGGAGCATT‐TAMRA‐3′ (10 μM stock; Integrated DNA Technologies)
  RPLP0 RT‐qPCR forward primer 5′‐TTGGGCATCACCACAAAGATT‐3′ (10 μM stock; Integrated DNA Technologies)
  RPLP0 RT‐qPCR reverse primer 5′‐CCCACTTTGTCTCCGGTCTTAA‐3′ (10 μM stock; Integrated DNA Technologies)
  RPLP0 Taqman Probe 5′‐FAM‐CATCACTCAGAATTTCAATGGTCCCTCGGG‐TAMRA‐3′ (10 μM stock; Integrated DNA Technologies)

20‐, 200‐, and 1000‐µl LTS pipet tips (Rainin, cat. nos. 17014961, 30389276, and 17014967)
1.5‐ml microcentrifuge tubes (Sarstedt, cat. no. 1081721)
Incubator, 37°C
Water bath, 42°C
15‐ and 50‐ml sterile conical polypropylene tubes (Falcon, cat. nos. 352097 and 352070)
5‐ml sterile serological pipets (Costar, cat. no. 4487)
10‐ml sterile serological plates (Sterilin, cat. no. M420800)
25‐ml sterile serological plates (Fisherbrand, cat. no. 13‐678‐11)
Rotating incubator, 37°C
Agarose gel electrophoresis apparatus
UV transilluminator
Surgical blades (Swann‐Mortor, BS2982)
Spectrophotometer (e.g., NanoDrop, Thermo Fisher Scientific)
Cell counting slides (Bio‐Rad, cat. no. 1450011)
Automated cell counter (Bio‐Rad, TC20)
NEPA electroporation cuvettes (NEPA GENE, cat. no. EC‐002S)
Electroporator (NEPA GENE)
Humidified culture incubator, 37°C ± 1°C, 5% ± 2% CO_2_ (Panasonic)
Microscope (Nikon‐TMS, cat. no. 310563)
0.5‐ml PCR tubes (Sarstedt, cat. no. 9084411)
Thermal cycler (G‐Storm)
6‐well plates, sterile
96‐well U‐bottom plates (Thermo Scientific, cat. no. 163320)
96‐well clear, flat‐bottom, polystyrene tissue culture plates, sterile (Thermo Scientific, cat. no. 167008)
Multichannel pipet, 20‐200 μl (Mettler Toledo, cat. no. 17013810)
Sterile reagent reservoirs (Star lab, cat. no. M0001636B0413)
Epifluorescence microscope (Leica DM IRB)
qPCR machine: e.g., QuantStudio^TM^ 5 (Applied Biosystems)

### Construction of full‐length segment A and segment B

1 Design the full‐length sequences of segments A and B of the IBDV strain of interest, including the 5′ and 3′ noncoding regions flanked with self‐cleaving ribozymes (a hammerhead ribozyme upstream and a hepatitis delta ribozyme downstream) and restriction enzyme sites (for example, KpnI‐HF and NheI‐HF). The constructs can be ordered from a gene synthesis service or company, for example GeneArt (Thermo Fisher Scientific), and typically arrive in a vector backbone, as lyophilized plasmids (pIBDV‐A and pIBDV‐B).
2 Reconstitute the pIBDV‐A and pIBDV‐B plasmids in 50 µl each of nuclease‐free water, and use them to transform competent *E. coli* by adding 1 µl of pIBDV‐A or pIBDV‐B to DH5α competent cells (50 µl) in a 1.5‐ml microcentrifuge tube and incubating for 30 min on ice.
3 Heat shock the bacteria for 45 s at 42°C and cool for 5 min on ice.
4 Add 450 µl SOC solution to the transformation mixture and incubate 1 hr at 37°C with shaking at 300 rpm.
5 Plate 100 μl of the mixture onto a LB agar plate supplemented with the antibiotic stipulated by the manufacturer and incubate overnight at 37°C.
6 Pick one colony and inoculate into 5 ml of LB broth supplemented with antibiotic in a 15‐ml polypropylene tube for 6 hr. Then transfer into 110 ml of LB broth supplemented with antibiotic in a conical flask.
7 Grow the bacteria overnight in a 37°C rotating incubator.
8 Isolate plasmids from the overnight culture using a maxi plasmid preparation kit following the manufacturer's protocol.
9 Digest the plasmids with restriction enzymes (for example, KpnI‐HF and NheI‐HF) according to the manufacturer's instructions.
10 Mix the restriction‐digested product with a loading dye. Load the sample and DNA ladder into wells of a 1% agarose gel and run the gel with TBE electrophoresis buffer.
11 Visualize the digested plasmids on a UV transilluminator: Positive clones will show two bands in each lane, one corresponding to the IBDV‐A (3.4 kb) or IBDV‐B (3.1 kb) and the other corresponding to the vector used by the gene synthesis company.
12 Cut out the bands corresponding to the IBDV‐A and IBDV‐B genes using a surgical blade. Use a separate, new surgical blade for IBDV‐A and for IBDV‐B.
13 Place the gel slice in a 1.5‐ml microcentrifuge tube and perform a gel extraction of IBDV‐A (3.4 kb) and IBDV‐B (3.1 kb) using a DNA purification kit following the manufacturer's instructions.
14 Determine the DNA concentration using a spectrophotometer.
15 Digest the expression vector pSF‐CAG‐KAN (2 µg) with KpnI‐HF and NheI‐HF restriction enzymes. pSF‐CAG‐KAN carries a kanamycin‐resistance gene.
  In general, perform restriction digestion at 37°C for 3 hr, and subsequent heat inactivation at 65°C for 20 min.
16 Mix the digest with loading dye, load the sample and DNA ladder on a 1% agarose gel and run the gel with TBE electrophoresis buffer. Confirm double digestion on the agarose gel by visualizing on a UV transilluminator, and cut out the digested bands (∼5.3 kb) using a surgical blade.
17 Purify the digested pSF‐CAG‐KAN vector using a DNA purification kit following the manufacturer's instructions. Determine the DNA concentration using a spectrophotometer.
18 Ligate the digested pSF‐CAG‐KAN vector with either the digested IBDV‐A (3.4 kb) or the digested IBDV‐B (3.1 kb), using T4 DNA ligase overnight at 4°C according to manufacturer's instructions, to prepare the reverse genetics (RG) plasmids: pRG‐IBDV‐A and pRG‐IBDV‐B.
  In general, the ligation mixture should include a 3:1 molar ratio of DNA fragment (240 ng) to plasmid DNA (80 ng).
19 The next day, transform *E. coli* by adding 5 µl of the pRG‐IBDV‐A or pRG‐IBDV‐B ligation mix to 50 µl DH5α competent cells in a 1.5‐ml microcentrifuge tube and incubating 30 min on ice. Heat shock for 45 s at 42°C and then cool for 5 min on ice. Add 450 µl SOC medium to the transformation mixture and incubate for 1 hr at 37°C with shaking.
20 Subsequently, plate the entire mixture (500 µl) on a LB agar plate supplemented with kanamycin and incubate overnight at 37°C.
  In general, we use 50 µg/ml kanamycin from the 50 mg/ml stock.
21 To screen for positive clones, pick individual colonies and inoculate into 5 ml LB broth supplemented with kanamycin in 15‐ml polypropylene tubes. Grow overnight in a 37°C rotating incubator.
22 Isolate plasmids from the overnight cultures using a mini plasmid preparation kit following the manufacturer's protocol and confirm pRG‐IBDV‐A‐ and pRG‐IBDV‐B‐positive clones by sequencing using the pSF‐CAG‐KAN vector forward primer 5′‐CTACCATCCACTCGACACACC‐3′ and reverse primer 5′‐GTTGTGGTTTGTCCAAACTCATCA‐3′.
23 Pick one positive colony for each of pRG‐IBDV‐A and pRG‐IBDV‐B, and inoculate into 5 ml of LB broth supplemented with antibiotic in a 15‐ml polypropylene tube for 6 hr. Then, transfer into 110 ml of LB broth supplemented with antibiotic into a conical flask.
24 Grow the bacteria overnight in a 37°C rotating incubator.
25 Isolate plasmids from the overnight culture using a maxi plasmid preparation kit following the manufacturer's protocol.

### Virus rescue by electroporation of pRG‐PBG98‐A and pRG‐PBG98‐B into DT40 cells

26 One hour before electroporation, prepare complete DT40 medium. Add 2 ml DT40 to each well of a 6‐well plate and place plate in the 37°C incubator.
  In general, keep a 6‐well plate with DT40 medium at 37°C for 1 hr to pre‐warm the medium.
27 For electroporation, prepare DT40 cells (1 × 10^7^ cells per well) in 100 µl Opti‐MEM medium in electroporation cuvettes in a biosafety cabinet.
  To count DT40 cells, we normally use cell counting slides and an automated cell counter. In general, after 3‐4 days of passaging, DT40 cells are suitable for electroporation.
28 Mix 10 µg each of pRG‐IBDV‐A and pRG‐IBDV‐B in in 100 µl Opti‐MEM.
29 Add the mixture of plasmids to the DT40 cells in Opti‐MEM.
30 Electroporate the mixture at 225 V with a pulse width of 2 ms poring pulse, and then transfer the electroporated mixture to 6‐well plate containing complete DT40 medium. Incubate for 48 hr in the humidified 37°C, 5% CO_2_ cell culture incubator.
31 At 48 hr after the electroporation, “feed” the cell cultures with fresh DT40 cells (∼1 × 10^6^ cells).
32 Every 72 hr, feed cultures with fresh DT40 cells in a 3:1 ratio of fresh to old cells.
33 After two passages, confirm the presence of rescued virus by fluorescence microscopy and RT‐qPCR.

### Confirmation of rescued virus in DT40 cells by fluorescence microscopy and RT‐qPCR

34 Harvest 500 µl (∼3 × 10^6^ cells) of the DT40 cell suspension, in duplicate aliquots. Prepare a DT40 cell pellet from one aliquot by centrifugation for 5 min at 1200 rpm. Then, extract RNA from a DT40 cell pellet, and perform virus quantification by RT‐qPCR (steps 49‐54). Confirm the presence of the recovered virus by immunofluorescence staining of another aliquot of cells using a primary antibody against IBDV and a secondary antibody conjugated to a fluorophore, and imaging with a fluorescence microscope (steps 36‐48).
35 After confirming virus recovery, continue to passage the recovered virus in DT40 cells in a 6‐well plate, and titrate to determine whether viral titers have increased.

#### Quantification of rescued virus in DT40 cells by immunofluorescence microscopy

36 Titrate the recovered virus using DT40 cells (1 × 10^5^ cells/well) in a 96‐well U‐bottom plate, and incubate the plate for 3 days at 37°C.
37 Add 180 µl DT40 cells (1 × 10^5^ cells/well) in complete DT40 medium into each well of a 96‐well U‐bottom plate.
38 Prepare a 10‐fold serial dilution of recombinant recovered virus in complete DT40 medium by adding 20 µl of the undiluted recombinant recovered virus to the first column of the plate, in quadruplicate.
39 Using a multichannel pipettor, mix the samples in each well at least five times, and then make a ten‐fold dilution of the samples by transferring 20 µl to the second column (i.e., moving left to right across the plate).
  Change the tip for each dilution pipetting step.
40 Repeat the dilutions from the second column to the third column, and continue until the last column. Discard 20 µl from the last column.
41 Incubate the plate for 3 days at 37°C.
42 After 3 days, remove the medium. Fix the cells in 4% paraformaldehyde diluted in PBS for 20 min at room temperature, permeabilize with 0.1% Triton X‐100 diluted in PBS for 10 min, and block with a 4% BSA solution for 60 min.
43 Incubate the cells with a primary antibody raised against IBDV, for example a mouse monoclonal antibody against the IBDV VP3 protein, for 1 hr at room temperature.
44 Wash the cells with PBS and incubate with a secondary antibody conjugated to a fluorophore, for example Alexa Fluor 488 or 568, for 1 hr at room temperature in the dark.
45 Wash cells and incubate for 10 min in DAPI solution.
  In general, we use a 1 in 10,000 dilution of DAPI in PBS.
46 Transfer cells to 96‐well clear, flat bottom plates to calculate viral titers or image cells.
47 Titrate or image cells using epifluorescence microscope such as a Leica DM IRB.
48 The highest dilution of the virus in which 50% of the wells show a VP3 signal is considered to be the endpoint, and the virus titer is calculated from the tissue culture infective dose‐50 (TCID_50_), according to the method of Reed and Muench, and expressed as TCID_50_/ml (Reed & Muench, 1938).

#### Quantification of rescued virus in DT40 cells by RT‐qPCR of rescued virus cell pellet in DT40 cells

49 Extract total RNA from the DT40 cell pellet (∼3 × 10^6^ cells) using an RNeasy kit according to the manufacturer's instructions.
50 Quantify the RNA samples using a NanoDrop or other spectrophotometer.
51 Perform reverse transcription of the RNA to generate cDNA using SuperScript III Reverse Transcriptase, following the manufacturer's instructions in regard to reaction constituents and conditions.
52 For virus quantification, perform qPCR using TaqMan^TM^ Universal qPCR Master Mix, according to the manufacturer's instructions.
53 Quantify IBDV replication by qPCR using IBDV primers and a Taqman probe with a qPCR machine, such as a QuantStudio^TM^ 5, with appropriate cycling conditions, for example: 95°C for 10 min followed by 40 cycles of 95°C for 15 s, 60°C for 1 min, 95°C for 15 s, 60°C for 1 min, 95°C for 30 s, and 60°C for 15 s (Dulwich et al., 2017, 2020).
54 Normalize target genes expression to a housekeeping gene, such as *RPLP0*, using primers and a Taqman probe and compare to the mock controls using the comparative delta C_T_ (2^–ΔΔCT^) method (Dulwich et al., 2017, 2020; Staines et al., 2016).

## Basic Protocol 2 A CHICKEN B‐CELL NEUTRALIZATION ASSAY FOR IBDV

**Figure 2:**
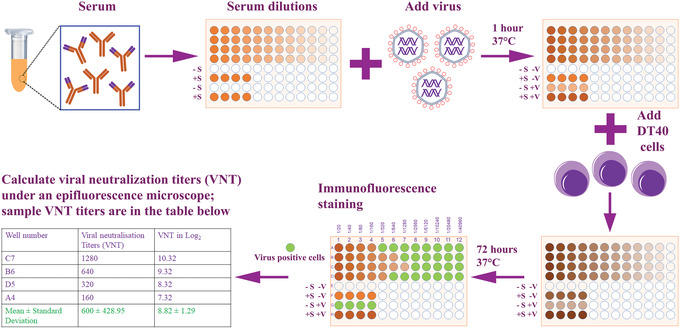
Overview of the chicken B‐cell neutralization assay for IBDV. Antibodies in polyclonal serum samples can be assessed for their neutralization capacity against diverse IBDV strains. After serial dilution of the heat‐inactivated serum samples, the virus to be tested is added at 10^3^ TCID_50_/ml and incubated for 1 hr at 37°C. DT40 cells are added to the serum/virus mix and the combination is incubated for 72 hr at 37°C. Cells are then fixed and stained with a monoclonal antibody against an IBDV antigen to identify wells that are positive or negative for IBDV, and finally the viral neutralization titers (VNT) are determined. Sample VNT titers are shown in the table. The figure was created using templates from the Motifolio toolkit (Motifolio Inc., Ellicott City, MD, USA).

This protocol describes an in vitro neutralization assay in the chicken B‐cell line DT40, to quantify the titer of antibodies present in polyclonal sera. Briefly, the IBDV is mixed with serial dilutions of heat‐inactivated sera and incubated at 37°C for 1 hr. Then, DT40 cells are added. After 3 days of culture, the infected cells are fixed and stained with an anti‐IBDV monoclonal antibody and secondary antibody conjugated to a fluorophore and wells are either scored or positive or negative for virus antigen and the virus neutralization titer (VNT) quantified. An outline of the neutralization assay is presented in Figure 2.

### Materials

Complete DT40 medium (see recipe)
Serum samples to be tested
Positive serum control samples with known neutralization activity
Virus stock to be tested
DT40 cells (chicken bursal lymphoma transformed B‐cell line; ATCC cat. no. CRL‐2111) grown in 75‐cm^2^ (T75) cell culture flasks (TPP, 90076, cat. no. 20210105)
0.4% trypan blue (Gibco, cat. no. 15250061)
Phosphate‐buffered saline (PBS)
Negative control (PBS)
15‐ and 50‐ml sterile conical polypropylene tubes (Falcon, cat. nos. 352097 and 352070)
5‐ml sterile serological pipets (Costar, cat. no. 4487)
10‐ml sterile serological plates (Sterilin, cat. no. M420800)
25‐ml sterile serological plates (Fisherbrand, cat. no. 1367811)
1.5‐ml microcentrifuge tubes (Sarstedt, cat. no. 1081721)
20‐ and 200‐µl LTS pipet tips (Rainin, cat. nos. 17014961 and 30389276)
Cell counting slides (Bio‐Rad, cat. no. 1450011)
Multichannel pipet, 20‐200 μl (Mettler Toledo, cat. no. 17013810)
Sterile reagent reservoirs (Star lab, cat. no. M0001636B0413)
96‐well U‐bottom plates (Thermo Scientific, cat. no. 163320)
96‐well clear, flat‐bottom, polystyrene tissue culture plates, sterile (Thermo Scientific, cat. no. 167008)
Automated cell counter (Bio‐Rad, TC20)
Humidified culture incubator, 37°C ± 1°C, 5% ± 2% CO_2_ (Panasonic)
Microscope (Nikon‐TMS, cat. no. 310563)
Epifluorescence microscope (Leica DM IRB)

### Prepare plates of serially diluted serum samples

1 Prepare the number of 96‐well U‐bottom plates required for the assay, and label the lids.
  Keep four controls on each plate: (i) positive serum and virus, (ii) positive serum only, no virus, (iii) virus only, no serum, and (iv) no serum, no virus. Set up all controls in quadruplicates.
2 Using a multichannel pipet, add 50 µl complete DT40 medium to each well in columns 2‐12.
3 Heat inactivate all the serum samples at 56°C for 30 min. Add 100 µl heat‐inactivated serum, diluted to 1:20 in complete DT40 medium, to the first column in quadruplicate.
4 Using a multichannel pipettor, mix the samples at least five times, and then make a two‐fold dilution of serum by transferring 50 µl from the first column to the second column.
  Change tips for every dilution pipetting step.
5 Repeat the dilutions from second column to third column, and continue until the last column. Discard 50 µl from the last column to make the volumes of plate equal.
6 Dilute an IBDV stock to a titer of 10^3^ TCID_50_/ml, and add 50 µl to each well of the serially diluted serum wells using multichannel pipet. Change tips after each virus adding step.
7 Include four controls, setting up each one in quadruplicate:
  - Positive serum and virus;
  - Positive serum only, no virus;
  - Virus only, no serum;
  - No serum, no virus.
    Positive serum are samples with known neutralization activity. Positive virus is presence of virus with titer of 10^3^ TCID_50_/ml.
8 Incubate the plates at 37°C for 1 hr.

### Calculation of DT40 cell number and addition to virus and serum mixture

9 Record the cell passage number and date of the most recent split DT40 cells in culture and grow cell stocks in T75 cell culture flasks.
  In general, 0.4% trypan blue is used for calculation of DT40 cell viability.
10 On the day of virus neutralization, dilute the DT40 cells to 1 × 10^6^ cells/ml in complete DT40 medium, and add 80 μl to each well (8 × 10^4^ cells per well) of a 96‐well U‐bottom plate using a multichannel pipet.
  Each plate requires ∼8 ml of cells.

### Calculation of neutralization titer

11 Incubate the plates at 37°C for 72 hr.
12 After 72 hr of incubation, follow the staining protocol described in Basic Protocol 1, steps 42‐45.
13 Transfer cells to 96‐well clear, flat bottom plates to calculate VNT.
14 Calculate VNT based on the highest dilution of serum where there are no IBDV antigen positive cells, and express as log_2_ value.
  VNT are calculated under an epifluorescence microscope, and sample VNT values are shown in the table in Figure 2. Sample VNT titers are shown in the table in Figure 2.

## REAGENTS AND SOLUTIONS

### Complete DT40 medium

300 ml RPMI 1640 medium (Corning, cat. no. 33720004)
50 ml 10% heat‐inactivated fetal bovine serum (FBS; Sigma‐Aldrich, cat. no. F2442)
50 ml tryptose phosphate broth (Gibco, cat. no. 18050039)
1 ml sodium pyruvate (Gibco, cat. no. 11360039)
500 µl of 50 mM 2‐mercaptoethanol (Gibco, cat. no. 31350010)
Dilute to 500 ml with more RPMI 1640
Store complete DT40 medium up to 3 months at 4°C.

## COMMENTARY

### Background Information

#### Basic Protocol 1: Chicken B‐cell rescue system for IBDV

Reverse genetics has been widely used to generate infectious molecular clones of viruses entirely from a full‐length cDNA copy of the viral genome. Reverse genetics systems for IBDV rely on the transfection of cells with two plasmids, one encoding IBDV segment A and one encoding segment B. In each plasmid, immediately upstream of the coding region and the 5′ noncoding region, is a self‐cleaving hammerhead ribozyme, and immediately downstream of the coding region and 3′ noncoding region is a self‐cleaving hepatitis delta ribozyme sequence. When cells are co‐transfected with both plasmids, VP1 is transcribed from segment B and translated, and VP2, VP3, VP4, and VP5 are transcribed from segment A and translated. In addition, the VP1 polymerase replicates the genome from the cleaved RNA sequence. Traditionally, immortalized cell lines, such as chicken embryo fibroblasts (CEFs) or DF‐1, QM7, or Vero cells, have been transfected with the plasmids to rescue cell‐culture‐adapted IBDV strains (Mendez et al., 2017; Mundt & Vakharia, 1996; Yao, Goodwin, & Vakharia, 1998; Zierenberg et al., 2004). In order to rescue a field strain of IBDV, researchers have in the past inoculated transfected CEFs or DF‐1 cell lysates into birds either intramuscularly (Nouen et al., 2012) or directly into the BF (Yu et al., 2012). Although these techniques successfully generated molecular clones of field strains 8818 and HLJ0504, respectively, they were laborious, required facilities for in vivo studies, and suffered from potential animal welfare issues. Recently, we and others demonstrated that field strains of IBDV can replicate within primary chicken bursal cells isolated from the BF and cultured in the presence of CD40L or phorbol 12‐myristate 13‐acetate (PMA) (Delgui, Gonzalez, & Rodriguez, 2009; Dulwich et al., 2017, 2018; Liu et al., 2019; Soubies et al., 2018; Terasaki et al., 2008). Furthermore, it was shown that the cell lysates from transfected DF‐1 cells could be passaged onto chicken primary bursal cells to rescue a molecular clone of a field strain (Cubas‐Gaona et al., 2020), which negated the need to inoculate chickens; but chickens were still needed to provide the primary bursal cells. The immortalized chicken B‐cell line DT40 has also been used to sustain IBDV field strains (Delgui, Gonzalez, et al., 2009; Terasaki et al., 2008), and here, we extend these observations by describing the rescue of a molecular clone of IBDV by electroporation of DT40 cells, bypassing the use of DF‐1 cells. Our laboratory recently used this protocol to rescue a panel of recombinant chimeric IBDVs containing the HVRs of diverse field strains in the backbone of the lab‐adapted strain PBG98 (Reddy et al., 2022).

#### Basic Protocol 2: Chicken B‐cell neutralization assay for IBDV

The quantification of serum antibodies against IBDV has traditionally been performed either by ELISA or through a neutralization assay in immortalized cells, such as DF‐1 cells (Huo et al., 2019; Ma et al., 2019), using a cell‐culture‐adapted viral strain, D78 (Sadigh et al., 2018). Therefore, cell‐culture‐adapted viruses are often used as a surrogate for the quantification of antibody responses elicited by infection with field strains or by vaccines containing the VP2 from field strains (Sadigh et al., 2018). Briefly, 100 TCID_50_ of cell‐culture‐adapted virus is mixed with heat‐inactivated, serially diluted serum and the mixture is added to adherent cells, such as DF‐1 cells. After 4 days, cultures are inspected for the presence or absence of cytopathic effect and the virus neutralization titer (VNT) is quantified. However, using this approach, it is not possible to quantify the VNT of vaccines against their target field strains, and it is not possible to quantify the cross‐neutralization VNT of antibodies elicited by one field strain against a heterologous field strain. To address this, the quantity of neutralizing antibodies can also be titrated in ovo in embryonated hens’ eggs, by mixing virus with serial dilutions of serum and inoculating the mixture into the eggs (Hitchner, 1971). The embryos are then inspected for signs of histopathology caused by the virus. Although this method can be used to evaluate neutralizing antibody responses against field strains of IBDV, it is laborious, as virus must be inoculated onto the chorioallantoic membrane (CAM) of each egg. The gold standard is to conduct cross‐protection studies in vivo by challenging birds that have serum antibodies against one field strain with a heterologous field strain to assess the level of protection, evaluated by the extent to which pathology is reduced. However, this requires considerable resources to complete successfully. As an alternative, here we describe the development of a neutralization assay using DT40 cells for the quantification of serum antibody responses against IBDV field strains. Moreover, the two protocols described can be combined by first generating a panel of recombinant viruses using Basic Protocol 1, and then conducting neutralization assays as per Basic Protocol 2. Our laboratory has recently used this approach to quantify the breadth of antibody responses elicited by an IBDV vaccine against a panel of recombinant chimeric IBDVs containing the HVRs of diverse field strains (Reddy et al., 2022).

## CRITICAL PARAMETERS AND TROUBLESHOOTING

### Basic Protocol 1: Chicken B‐cell Rescue System for IBDV

*Construction of full‐length segment A and segment B*. Check that the correct size of insert has been cloned into the pSF‐CAG‐KAN vector (IBDV‐A: 3.4 kb; IBDV‐B: 3.1 kb) and confirm the identity of the insert by Sanger sequencing.

*Virus rescue by electroporation of pRG‐PBG98‐A and pRG‐PBG98‐B into DT40 cells*. To obtain good electroporation efficiency, it may be necessary to optimize the electroporation voltage, within a range of 150‐300 V, and the time of electroporation, within a range of 1‐5 ms.

*DT40 cells*. Healthy DT40 cells are important for the electroporation; therefore, you should usually split the cells at least two or three times after thawing them from the liquid nitrogen.

### Basic Protocol 2: Chicken B‐cell Neutralization Assay for IBDV

*DT40 cell‐density calculation and addition of the cells to virus and serum mixture*. Healthy DT40 cells are important for the neutralization assay; therefore, split the cells at least two or three times after thawing from the liquid nitrogen. Include suitable positive and negative controls for serum and virus included in every experiment.

## UNDERSTANDING RESULTS

### Chicken B‐Cell Rescue System for IBDV

The presence of viral‐antigen‐positive cells after two passages in DT40 cells is indicative of virus rescue, and a subsequent increase in viral titer with passaging verifies virus rescue.

### Chicken B‐Cell Neutralization Assay for IBDV

The final neutralization titers are calculated by measuring the lowest concentration of serum at which there viral antigen is absent from the cells in the wells, consistent with the virus being neutralized by the serum (the presence of viral‐antigen‐positive cells indicates that the virus has not been neutralized). The neutralization titers are expressed as log_2_ values and the mean titers of the quadruplicate wells are calculated.

## TIME CONSIDERATIONS

### Chicken B‐Cell Rescue System for IBDV

Synthesis of IBDV‐A and IBDV‐B through gene synthesis services may take several weeks. Cloning of the IBDV‐A and IBDV‐B genes into the pSF‐CAG‐KAN vector can take 2 weeks. Electroporation and recovery of the virus in DT40 cells can take up to 2 weeks, and each subsequent passage takes ∼3‐4 days. Titration of rescued virus supernatant in DT40 cells requires 4‐5 days, and RT‐qPCR requires 3‐4 days.

### Chicken B‐Cell Neutralization Assay for IBDV

The neutralization assay plate setup, staining, and calculation of the neutralization titers can take up to 4‐5 days.

### Author Contributions

**Vishwanatha Reddy**: Conceptualization, data curation, formal analysis, investigation, methodology, project administration, resources, software, supervision, validation, visualization, writing—original draft, writing—review and editing. **Salik Nazki**: Formal analysis, methodology, validation. **Amin Asfor**: Methodology, validation. **Andrew Broadbent**: Conceptualization, funding acquisition, project administration, resources, writing—original draft, writing—review and editing.

### Conflict of Interest

The authors declare no conflict of interest.

## Data Availability

Data sharing not applicable—no new data were generated.
